# In Vitro Functional Properties of Rosehips from ‘Aurora’ Edible Garden Rose’s Collection

**DOI:** 10.3390/foods13203272

**Published:** 2024-10-15

**Authors:** Nemanja Živanović, Biljana Božanić Tanjga, Nataša Simin, Marija Lesjak, Bojana Blagojević, Magdalena Pušić Devai, Mirjana Ljubojević, Tijana Narandžić

**Affiliations:** 1 Faculty of Sciences, University of Novi Sad, Trg Dositeja Obradovića 3, 21000 Novi Sad, Serbia; 2 Breeding Company ‘Pheno Geno Roses’, Maršala Tita 75, 23326 Ostojićevo, Serbia; 3 Faculty of Agriculture, University of Novi Sad, Trg Dositeja Obradovića 8, 21000 Novi Sad, Serbia

**Keywords:** rosehips, *Rosa* × *hybrida*, polyphenols, antioxidants, vitamin C, acetylcholinesterase

## Abstract

Although they have been extensively studied in many species of the genus *Rosa* L., garden roses’ hips have largely been overlooked. To investigate their potential use in the food industry, this study evaluated five cultivars from ‘Aurora’ collection: ‘Purple Aurora’, ‘Berry Bush Aurora’, ‘Aromatic Aurora’, ‘Butterfly Aurora’, and ‘Rugose White Aurora’. Morphological characterization, along with the assessment of the phenolic profile, vitamin C levels, and biological activities—including antioxidant and neuroprotective effects—was conducted. The fruit mass reached 5.15 g, while the mesocarp mass ranged from 3 to 4 g, resulting in a mesocarp-to-fruit ratio of over 75%. The total phenolic content ranged from 37.1 to 63.9 mg GAE/g de, while total flavonoids were present in amounts from 0.85 to 2.14 mg QE/g de. Rosehip extracts from four out of five cultivars exhibited a very high vitamin C content, reaching 2384 µg/g fw. Quinic acid and 31 phenolic compounds were found in the rosehip extract of at least one cultivar. Results indicated ‘Aurora’ rosehips have potent antioxidant properties and a moderate inhibitory effect on acetylcholinesterase, highlighting their potential as a source of functional food. Additional research is needed to fully leverage these benefits and establish garden rosehips as a viable alternative to synthetic antioxidants.

## 1. Introduction

Roses, classified under the family Rosaceae and the genus *Rosa*, represent a diverse plant group, with over 30,000 recognized cultivars currently documented. They have a long tradition of cultivation not only for decorative purposes but also for the production of rose essential oil, rose water, and rose concrete, and their attractive colors and aromas have made them popular ingredients in various foods, including drinks, salads, and desserts [1,2,3,4]. Moreover, roses produce a pseudo-fruit known as the rosehip, which has a long history of use in various culinary applications, including teas, jams, jellies, and juices, as well as in traditional national dishes. In addition to their aromatic qualities, rosehips are valued for their medicinal properties. They have been used in traditional medicine to treat a range of conditions, including the common cold, influenza, gastrointestinal disorders, and inflammation [5,6].

Rosehips are recognized for their significant nutritional value, being particularly rich in essential nutrients such as vitamins A, B complex, E, and notably vitamin C, as well as fatty acids, carotenoids, triterpenoids, and polyphenols. These compounds are well-documented for their beneficial effects on human health [7]. Research has demonstrated that rosehips from various species, including well known *Rosa canina* L., as well as *Rosa moschata* Herrm., *Rosa dumalis* Bechst., *Rosa rubiginosa* L., *Rosa vilosa* L., and hybrids of *R. vilosa*, contain substantial amounts of polyphenols, vitamin C, and carotenoids. Notably, they exhibit greater antioxidant capacity compared to other nutrient-dense fruits, such as blackcurrants, blueberries, strawberries, red raspberries, and sea buckthorn [8]. Phenolic compounds are primarily concentrated in the skin of the rosehips, with lower concentrations found in the pappi and the seeds. Given that whole rosehips are utilized as a dietary component, it is essential to assess the overall nutrient composition of the entire rosehip fruit [9,10]. Furthermore, rosehips are known to contain various organic acids, including quinic, malic, and citric acids, as well as several polyphenolic acids, such as *p*-coumaric, chlorogenic, gallic, protocatechuic, rosmarinic, caffeic, ferulic, and vanillic acids. In addition, they contain flavonoids such as catechin, quercetin, rutin, myricetin, hyperoside, and isoquercetin. These compounds have been identified in species including *R. canina*, *Rosa arvensis* Huds., *R. dumalis* Bechst., *Rosa dumetorum* Thuill., and *Rosa sempervirens* L. [5,11,12,13].

Beneficial effects of rosehip extracts have been confirmed in various in vivo studies. Specifically, extracts from *R. canina*, along with tiliroside—a flavonoid glycoside that is a major component of rosehip seeds—have been shown to reduce lipid accumulation in a high-fat diet-induced obesity model in mice [14]. This finding was further supported by a clinical trial [15]. Additionally, hot water extracts of rosehips exhibited a protective effect against diabetes in spontaneously diabetic Torii rats [16]. Furthermore, extracts from *R. canina* demonstrated a significant antiproliferative effect on glioblastoma cells, exceeding the efficacy of Temozolomide, a widely used chemotherapeutic agent for glioblastoma treatment [17]. Rosehips also exhibit notable anti-inflammatory properties [18].

Today, there is an increasing trend toward creating foods with natural aromas and colors that offer additional nutritional or functional benefits and replace synthetic antioxidants [19]. Also, there is a growing trend of incorporating garden roses into culinary applications, including in Michelin-starred restaurants. Chefs are increasingly exploring the use of edible flower parts for their unique flavors, colors, and aesthetic appeal. Garden roses, particularly their panels, are being used in various dishes, ranging from desserts to savory courses, as well as in beverages. Thus, consumers are increasingly drawn to these products, driving higher demand.

Rosehips, known for their pleasant aromas and rich chemical composition, are emerging as an excellent candidate for use in functional food products [20,21,22,23]. However, the content of biologically active compounds in rosehips can vary significantly due to genetic, physiological, and environmental factors [24]. Furthermore, research has predominantly focused on rosehips from *R. canina*, commonly known as dog rose, while rosehips from other species, such as the garden rose (*Rosa × hybrida* L.), have been largely overlooked. Therefore, further evaluation of rosehips from various species and regions is necessary to fully assess their potential value in the functional food industry.

To enhance the understanding of the potential use of garden roses in the food industry, this study aimed to evaluate rosehips from new garden rose cultivars cultivated in the Vojvodina region of Serbia. Specifically, the research focused on five garden rose cultivars from the ‘Aurora’ collection, assessing their morphological characteristics, phenolic profile, vitamin C levels, and biological activities, including antioxidant potential and inhibition of acetylcholinesterase (AChE). This study represents the first comprehensive investigation of the chemical profile and beneficial effects of rosehips from garden roses, both globally and within the Balkan region, which is renowned for its high-quality Bulgarian roses.

The investigated cultivars demonstrated resistance to drought, heat, and diseases. When grown on their own roots, they also exhibited significant tolerance to poor-quality, saline-rich, and heavy soils. With their robust, bushy growth habit and capacity to produce abundant flowers year-round, these cultivars are increasingly utilized in landscaping [25].

## 2. Materials and Methods

### 2.1. Plant Material

The plant material used in the experiments included rosehips from five garden rose cultivars from ‘Aurora’ collection: ‘Purple Aurora’ (PAU), ‘Berry Bush Aurora’ (BBA), ‘Aromatic Aurora’ (ARA), ‘Butterfly Aurora’ (BA), and ‘Rugose White Aurora’ (RWA). The cultivars were developed by Pheno Geno Roses from Serbia and were grown on their experimental field in Temerin, Northern Serbia (45°24′19″ N 19°53′13″ E). The region experiences a continental climate with cold winters and warm summers. The trial, initiated in autumn 2020, involved on-site bud grafting onto *Rosa laxa* Retz. The plants were arranged 10 cm apart within rows and 1 m between rows, and no irrigation or chemical treatments were applied.

### 2.2. Morphological Characterization

**Figure 1:**
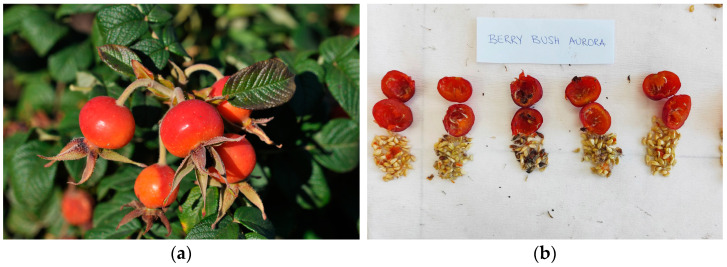
Fruits of the cultivar ‘Berry Bush Aurora’ (BBA) were evaluated for quantitative and qualitative features: (**a**) rosehips in the field; (**b**) the assessment of fruit, mesocarp, and seed traits in the laboratory.

The morphological assessment of rosehips was conducted in October 2023, with 25 replications per cultivar. The assessment included both metric and organoleptic parameters. Measurements and calculations were made for fruit mass (g), height, width, and thickness (mm), mesocarp mass (g), mesocarp thickness (mm), seed number per fruit, seed mass (g), and mesocarp-to-fruit ratio (Figure 1). Rosehips were also evaluated for fruit shape, color, mesocarp color, and taste. The morphological characterization followed the UPOV protocol for roses (*Rosa* L.) [26]. Qualitative assessment was conducted by three independent researchers to minimize subjectivity. Considering the current regulations for ethics in food science research, we collected informed consent, while assuring data protection and privacy maintenance.

### 2.3. Rosehip Extract Preparation

Rosehips were collected during October 2023 and kept frozen until the study was conducted. Frozen rosehips were grounded in a mortar with a pestle, spread on Petri dishes, and dried to constant mass in an air drier at 40 °C. Extraction was performed according to the procedure laid out by Šibul et al. [27]. Dry rosehips were macerated with 80% MeOH (1:10 ratio) for 2 h, at room temperature and on a stirrer (160 rpm). After 2 h, extracts were filtered, and maceration was repeated 2 more times. Macerates were evaporated to dryness in vacuo at 40 °C, dry extracts were dissolved in DMSO to the final concentration of 200 mg/mL. These extracts were used for the determination of total phenolic content (TPC), total flavonoid content (TFC), HPLC analysis, and examination of biological activities, such as antioxidant by 2,2-diphenyl-1-picrylhydrazyl (DPPH) and ferric reducing antioxidant potential (FRAP) assays, and the potential to inhibit AChE.

Extraction of Vit C was performed using a procedure described by Hernández et al. [28] with some modifications. Frozen rosehips were cut with a knife into small pieces and these were put in a 50 mL plastic tube. Extraction was performed with 3% metaphosphoric acid (MPA) cold solution (1:5 ratio) and homogenized by IKA^®^ T18 basic ultra-turrax^®^ (IKA-Werke GmbH & Co. KG, Staufen, Germany) for 30 s; after this, extracts were centrifuged (10 min, 9000× *g*, 4 °C), filtered, and filled to the volume of 50 mL with 3% MPA in a measuring flask. Determination of Vit C content in the obtained extracts was performed on the same day.

### 2.4. Determination of TPC

TPC was determined by using Folin–Ciocalteu (FC) reagent according to the method described by Simin et al. [4]. Sample concentrations were 0.25, 0.5, and 1.0 mg/mL. All tests were performed in triplicate and the total phenolic content was expressed as mg of gallic acid equivalents per g of dry extract (mg GAE/g de) or mg of gallic acid equivalents per g of fresh weight (mg GAE/g fw).

### 2.5. Determination of TFC

TFC was determined by a colorimetric method described in Simin et al. [4]. Sample concentrations were 5, 10, and 20 mg/mL. All tests were performed in triplicate and results were expressed as mg of quercetin equivalents per g of dry extract (mg QE/g de) or µg of quercetin equivalents per g of fresh weight (µg QE/g fw).

### 2.6. Quantitative Analysis of Selected Phenolic Compounds by LC-MS/MS

The content of quinic acid and 44 selected phenolic compounds (14 phenolic acids, 25 flavonoids, 3 coumarins and 2 lignans) was investigated by liquid chromatography with tandem mass spectrometry (LC-MS/MS) according to the previously reported method [29]. Standards of the compounds were purchased from Sigma-Aldrich Chem (Steinheim, Germany), Fluka Chemie GmbH (Buchs, Switzerland), or from ChromaDex (Santa Ana, CA, USA). Samples and standards were analyzed using Agilent Technologies 1200 Series high-performance liquid chromatograph coupled with Agilent Technologies 6410A Triple Quad tandem mass spectrometer with electrospray ion source, and controlled by Agilent Technologies MassHunter Workstation software—Data Acquisition (ver. B.04.00) (Agilent Technologies, Inc., Santa Clara, CA, USA). All extracts were diluted with 50% aqueous MeOH to the concentrations of 20 mg/mL. The sample (5 μL) was injected into the system, and compounds were separated on a Zorbax Eclipse XDB-C18 (50 mm × 4.6 mm, 1.8 μm) rapid-resolution column. Data were acquired in dynamic Multiple Reaction Monitoring (MRM) mode. Peak areas were determined using Agilent MassHunter Workstation Software—Qualitative Analysis (ver. B.06.00). Calibration curves were plotted by OriginLabs Origin Pro (ver. 2019b) software and used for calculating the investigated compound concentrations in the extracts. Retention times, MS parameters (precursor ion *m*/*z*, product ion *m*/*z*, fragmentor and collision energy), and standard curve equations and coefficients of determination are given in the Supplementary Materials for the compounds that were quantified in the rosehip methanol extracts (Table S1).

### 2.7. Determination of Vit C Content

The content of Vit C was determined according to Simin et al. [4] with small modifications. Briefly, 270 μL of 72 mg/L dichlorophenolindophenol (DCPIP) reagent was mixed with 30 μL of extract (diluted with distilled water 1:1) or standard solution (ascorbic acid in the range of concentrations from 5 to 320 µg/mL in 1.5% MPA) and after 5 min of incubation, the absorbance was measured at 515 nm. In the blank probe, DCPIP reagent was substituted with dH_2_O. All tests were performed in triplicate and the content of vitamin C was expressed in µg per g of fresh weight (µg/g fw).

### 2.8. Antioxidant Potential

#### 2.8.1. DPPH Assay

The ability of the extracts to neutralize DPPH^•^ was determined according to Simin et al. [4]. Samples were tested in the concentration range 0.078–5.0 mg/mL. All tests were performed in triplicate and the results were expressed as IC_50_ value (the extract concentration that neutralizes 50% of DPPH^•^ (µg/mL)).

#### 2.8.2. FRAP Assay

FRAP assay was performed according to Simin et al. [4]. Sample concentrations were 0.25, 0.5, and 1.0 mg/mL. All tests were performed in triplicate and the results were expressed as mg of ascorbic acid equivalents per g of dry extract (mg AAE/g de).

### 2.9. Inhibition of AChE

The potential to inhibit AChE was assessed using Ellman’s method with certain modifications as previously described in Simin et al. [4]. Samples were tested at a concentration of 10 mg/mL. All tests were performed in triplicate and the results were expressed as percent of inhibition.

### 2.10. Statistical Analysis

The data of all spectrophotometric measurements were analyzed by one-way ANOVA followed by the post hoc Tukey’s honest significant difference (HSD) test for multiple comparisons of means to determine whether the data obtained for different rose cultivars differed significantly between each other (Real Statistics Resource Pack add-in for Excel 2013, Microsoft Corporation, Redmond, WA, USA). Statistical significance was set at *p* ≤ 0.05. Correlation factors between chemical composition and in vitro biological activities were calculated using regression analysis in Excel 2013.

## 3. Results

### 3.1. Morphological Features of Rosehips

**Figure 2:**
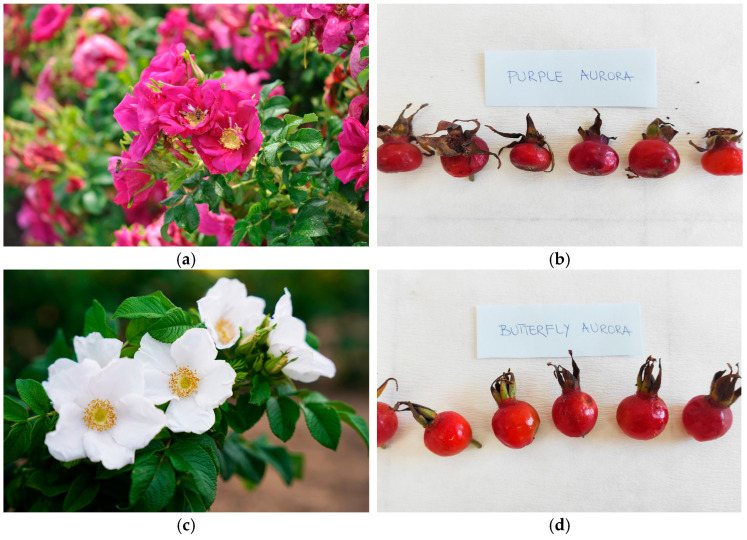
Two cultivars with contrasting fruit mass, dimensions, and flatness level: (**a**,**b**) ‘Purple Aurora’ (PAU) plant in the field and harvested rosehips; (**c**,**d**) ‘Butterfly Aurora’ (BA) plant in the field and harvested rosehips.

Among the investigated rose cultivars, notable differences in rosehip morphological traits were observed (Table 1). Fruit mass ranged from 3.87 g in PAU rosehips to 5.15 g in BA rosehips (Figure 2). Statistically significant differences were also found in fruit dimensions, with PAU and RWA showing the smallest values: an average height of 12.85 and 14.22 mm, respectively, and width and thickness below 18 mm. The largest rosehips were from the BA cultivar, with a height exceeding 27 mm and width and thickness above 23 mm. Mesocarp mass ranged from 3 to 4 g, accounting for more than 75% of the total fruit mass in all assessed cultivars. The lowest number of seeds per fruit was found in RWA and ARA (below 40 seeds per rosehip), while the highest seed number was observed in BBA (average of 56.17).

All investigated cultivars had pitcher-shaped rosehips, with varying proportions of flattened fruits among the total number of evaluated rosehips per cultivar (Table 2). In PAU, all fruits were flattened, whereas BA rosehips lacked this characteristic entirely (Figure 2). Other cultivars exhibited varying proportions of flattened rosehips. Fruit color ranged from red to orange, and the mesocarp was consistently orange across all cultivars. The fruits did not differ significantly in taste, being characterized as moderately acidic and moderately sweet, regardless of the level of fruit flattening, shape, or color.

### 3.2. Chemical Profile of Rosehip Extracts

Determination of TPC, TFC, and Vit C in the rosehip methanol extracts indicated significant differences among investigated cultivars (Table 3). The TPC ranged from 37.1 mg GAE/g de in RWA to 63.9 mg GAE/g de in BBA. When expressed per gram of fresh weight, the highest TPC was found in PAU and BBA rosehips, reaching 7.72 and 6.45 mg GAE/g fw, respectively. The results of TFC assessment in examined cultivars showed that TFC values were notably lower than obtained TPC values, indicating that flavonoids represent only a small portion of total phenolic compounds present in investigated rosehips. The highest TFC was determined in PAU (2.14 mg QE/g de), followed by BBA and RWA, with values of 1.80 and 1.70 mg QE/g de, respectively. When expressed per g of fresh weight, TFC varied from 82.1 µg QE/g fw in ARA to 296 µg QE/g fw in PAU.

A very high content of Vit C, above 2000 µg/g fw, was found in rosehip extracts of four out of five cultivars. The ARA exhibited the highest content of Vit C, at 2384 µg/g fw, while values obtained for other cultivars were decreasing in the following order: PAU > BBA > BA > RWA. In RWA, significantly lower content was found, reaching only 218 µg/g fw.

In addition to the analysis of TPC, the presence of quinic acid and 44 phenolic compounds was quantified using LC-MS/MS (Table 4). Out of them, quinic acid and 31 phenolic compounds were detected in the rosehip extract of at least one cultivar, while the remaining 13 analyzed compounds were not detected in any cultivar.

A high content of non-phenolic intermediate in the biosynthesis of phenolic compounds—quinic acid, was determined in all analyzed samples, ranging from 85.9 μg/g de in ARA cultivar, to 176 μg/g de in PAU. Among detected hydroxybenzoic acids, gallic acid was present in the highest quantities, ranging from 28 μg/g de found in PAU to 63.4 μg/g de, determined in RWA. Besides gallic, both *p*-hydroxybenzoic and protocatechuic acids were detected in all cultivars, but in much lower quantities (<5.7 μg/g de). Gentisic and syringic acids were also found in some samples. Regarding the detected hydroxycinnamic acids, four out of five were determined in all examined extracts. Among them, *p*-coumaric acid was the dominant one, the content of which ranged from 14.5 μg/g de in RWA to 46.7 μg/g de in BBA. Other hydroxycinnamic acids detected were caffeic, ferulic, sinapic, and chlorogenic acids, which content did not exceed 10.6 μg/g de. The content of coumarin esculetin was within the range of 0.15–0.44 μg/g de. Due to the overlapping peaks of quercetin 3–*O*–glucoside and quercetin 3–*O*–galactoside in the chromatogram, only their total amount (quercetin 3–*O*–Glc + Gal) could be quantified, ranging from 234 μg/g de in PAU to 367 μg/g de in BBA. Beside them, predominant flavonols in all investigated samples were quercetin (48.5–124 μg/g de), quercitrin (29.2–73 μg/g de), rutin (12.4–64.2 μg/g de), and kaempferol 3–*O*–Glc (37.9–46.8 μg/g de). Six out of nine detected flavons were present in all cultivars in low quantities, including amentoflavone, luteolin, luteolin 7–*O*–Glc, apigenin, apigenin 7–*O*–Glc, and chrysoeriol. Flavanone naringenin was found in the highest amount in BBA and BA cultivars (6.78 and 6.61 μg/g de, respectively, while the highest content of flavanol epicatechin was determined in PAU (5.84 μg/g de). Another flavanol, catechin, reached significantly high quantities in PAU and BBA (347 and 279 μg/g de, respectively), while the lowest amount of this compound was detected in RWA (43 μg/g de).

The total content of all detected compounds using LC-MS/MS expressed in mg/g de, was the highest in BBA and PAU rosehip extracts. Compounds not found in any of the investigated samples were as follows: cinnamic acid, *o*–coumaric acid, umbelliferon, vanillic acid, scopoletin, 3,4–dimethoxy cinnamic acid, daidzein, genistein, baicalein, epigallocatechin gallate, matairesinol, secoisolariciresinol, and baicalin. Chromatograms are given in the Supplementary Materials, Figures S1–S5.

### 3.3. Antioxidant Activity and Potential of Rosehip Extract to Inhibit AChE

Rosehip extracts showed high antioxidant activity for all analyzed cultivars (Table 5). The strongest antioxidants were the extracts of BBA, PAU, and BA. Those extracts reached IC_50_ values in DPPH assay of 34.2, 34.5, and 37.4 µg/mL, followed by antioxidant potentials in the FRAP assay of 68.5, 52.6, and 56.6 mg AAE/g de, respectively. Significantly lower antioxidant activity was determined for ARA, while the extract of RWA exhibited the lowest antioxidant activity, with an IC_50_ value in the DPPH assay of 115 µg/mL and an antioxidant potential in the FRAP assay of 37.5 mg AAE/g de.

The obtained results show the potential of rosehip extracts of the investigated cultivars to inhibit AChE at a concentration of 0.5 mg/mL (Table 5). The determined differences in the potential of the rosehip extracts to inhibit AChE were not statistically significant, with the exception of ARA, which extract expressed the lowest level of investigated activity (29.6%). For the extracts of the remaining four cultivars, the percentage of inhibition ranged from 37.2% for BA to 40.4% for the RWA cultivar.

### 3.4. Correlation Analysis

The correlation between the chemical composition of rosehip methanol extracts and in vitro biological activities was determined by regression analysis; the correlation factors (r^2^) are given in Table 6.

There is a high level of correlation between chemical composition and antioxidant activity. This is not surprising considering that Vit C and phenolic compounds are good natural antioxidants. Antioxidant activity determined by DPPH assay have been shown to have high correlation with Vit C, TPC, TSC, quinic acid, and quercitrin. On the other hand, TPC, TFC, TSC, quinic and *p*-coumaric acid, quercetin, quercetin 3–*O*–Glc + Gal, quercitrin, and kaempferol 3–*O*–Glc contents were highly correlated with antioxidant activity, determined by FRAP assay.

When it comes to the inhibition of AChE, high levels of correlation were shown by TPC, TFC, and TSC, as well as the contents of quinic acid, gallic acid, quercetin 3–*O*–Glc + Gal, quercitrin, and kaempferol 3–*O*–Glc.

## 4. Discussion

In addition to their extensive use in landscaping, roses are emerging as a novel source of functional food, offering a range of benefits for the food industry in both fresh and processed forms. However, garden roses, particularly their rosehips, have not been thoroughly evaluated for their quality as functional food ingredients. To our knowledge, there are currently no available data on the phenolic profile and biological activities of rosehips from garden roses, either globally or specifically from the Balkan region, which is renowned for its Bulgarian rose cultivars [1,4,30]. This study aims to address this gap in the knowledge by assessing the potential of garden rose rosehips grown in Serbia as valuable raw materials for the functional food industry. Additionally, it provides insights into the morphological traits of the investigated rose cultivars.

The desirable morphological features of rosehips primarily relate to their mass, dimensions, and the mesocarp-to-fruit ratio. This study showed that rosehips from the ‘Aurora’ collection included cultivars with a fruit mass of about 4 to 5 g, with the mesocarp accounting for 75.47% to 79.5% of the total fruit mass. These results highlight the high value of the investigated rosehips for the food processing industry, compared to our previous findings and those of other authors. Previous research by Božanić Tanjga et al. [3] on the ‘Mella’ garden rose collection reported similar metrics, with hip lengths up to 18 mm and widths reaching 15 mm; however, some cultivars in the present study had significantly larger hip dimensions. Bozhuyuk et al. [31] found that unsprayed genotypes of *R. canina* and *R. dumalis* had rosehips weighing between 2.95 g and 4.72 g, with a fruit flesh ratio of 62.55% to 74.42%, which was considerably lower than that of ‘Aurora’ rosehips. Ubaydullayev and Gaffarov [32], in their assessment of 32 varieties from different rose species, found fruit lengths, diameters, and weights ranging from 19 to 35 mm, 14 to 21 mm, and 2.3 to 6.4 g, respectively, with fruit flesh proportions varying from 58.3% to 85.2%. Ercisli and Guleryuz [33] identified promising varieties with mesocarp-to-fruit ratios of 61.67–74.20%, supporting our findings. As an additional factor in determining economically viable rose cultivars, seed numbers per rosehip did not show significant differences among the investigated rosehips, ranging from 35.18 to 56.17 seeds on average. Türkben et al. [34] found 11.0 to 35.3 seeds per rosehip, though these were associated with lower fruit weights (0.88 to 2.22 g) compared to those in our study. In the research by Ubaydullayev and Gaffarov [32], seed numbers per fruit ranged from 8.8 to 36.3, but the seeds were notably heavier (0.02 to 0.06 g) than those from ‘Aurora’ cultivars. Considering the need for larger fruit mass and dimensions, a higher flesh proportion, and a lower seed number per rosehip, the BA cultivar exhibited the most promising morphological traits. Regarding the organoleptic properties of the rosehips, the same cultivar stood out as the only one with non-flattened fruits, which could be advantageous for fruit utilization and easier processing.

According to Kunc et al. [35], the most important phenolic compounds detected in rosehips are tannins, flavonoids, phenolic acids, and anthocyanins. However, the phytochemical characteristics of rosehips vary due to factors such as genotype, cultivar, growing region, environmental conditions, growth practices, harvest time, and ripening stage [36,37,38]. This study found variability in TPC and TFC values among different cultivars, aligning with previous research. For instance, Bozhuyuk et al. [31] reported TPC values from 3.90 to 5.19 mg GAE/g fw in *R. canina* and *R. dumalis*. Similarly, Karataş [39] found TPC values ranging from 10.18 to 14.07 mg GAE/g fw in various *Rosa pimpinellifolia* genotypes. In another study, Sagbas [40] observed TPC values from 4.70 to 6.44 mg GAE/g fw in *R. canina* ecotypes, and Mertoğlu et al. [41] reported an average TPC of 3.73 mg GAE/g for the same species. The cultivars assessed in this study showed comparable or higher TPC values, except for content noted in *R. pimpinellifolia* [39], highlighting the substantial levels of these bioactive compounds in rosehips, particularly in PAU and BBA. Notably, TFC content, which constitutes a smaller proportion of total phenolics, also identified the PAU cultivar as having the highest values of 2.14 mg QE/g de and 296 µg QE/g fw. Interestingly, this cultivar also exhibited the smallest fruit mass and mesocarp thickness among the cultivars studied, with a mesocarp-to-fruit ratio of 76.89%. *Rosa canina* ecotypes evaluated by Sagbas [40] exhibited varying levels of TFC, ranging from 1.18 to 2.64 mg QE/g. In comparison, Bozhuyuk et al. [31] reported TFC values between 0.88 and 2.04 mg QE/g fw, indicating much lower quantities of flavonoids in the rosehips of the ‘Aurora’ collection and highlighting the need for further investigation of other phenolic compounds potentially present in these fruits.

Among the five cultivars analyzed, four (PAU, BBA, ARA, and BA) exhibited high Vit C content (in the range 201.1–238.4 mg/100 g fw). In contrast, lower Vit C levels, ranging from 37 to 53 mg/100 g fw, were found in *R. pimpinellifolia* [38]. Slightly higher values were recorded in rosehips grown in Romania, ranging from 112 to 360 mg/100 g fw [42]. Sicilian rosehip species showed amounts between 222 and 513 mg/100 g fw [43], while significantly higher Vit C content, up to 2557 mg/100 g fw, was found in Turkish native species by Ercisli and Esitken [44]. These findings indicate that rose cultivars, including garden roses, are a valuable source of Vit C, present in rosehips at much higher quantities compared to other fruits, such as apples, oranges, and strawberries, which contain 6, 46, and 61 mg/100 g, respectively, as reported by Proteggente et al. [45].

From the compounds targeted for quantification and detected in rosehip methanol extracts, Demir et al. [46] identified several phenolic acids in all analyzed rosehip extracts, including gallic acid, *p*-hydroxybenzoic acid, chlorogenic acid, *p*-coumaric acid, and ferulic acid. In contrast, they detected epicatechin gallate in only one species, *R. dumalis* subsp. *boissieri*, whereas in our study, this compound was absent in all cultivars. Gallic acid was present in the highest quantities among all detected hydroxybenzoic acids in our study, ranging from 28 to 63.4 μg/g de, whereas Demir et al. [46] recorded a maximum value of only 12.93 μg/g de in *Rosa hirtissima*. Similar levels of caffeic acid were observed. *p*-Coumaric acid, present in the highest amounts among detected hydroxycinnamic acids, was also found in significantly higher quantities compared to Demir et al.’s results. Bozhuyuk et al. [31] also detected chlorogenic acid, followed by gallic acid, *p*-coumaric acid, and caffeic acid in *R. canina* and *R. dumalis* rosehips, with much lower gallic acid content compared to our results, reaching up to 49.3 μg/g fw. In contrast, chlorogenic and caffeic acids were present in lower amounts in rosehip extracts from the ‘Aurora’ collection compared to up to 81.3 μg/g fw and 14.2 μg/g fw detected in the referenced study, respectively. In their investigation of rosehip species distributed in China, Sun et al. [47] found that more than 50% of the total detected compounds were phenolic acids and flavonoids. Among the phenolic acids, they identified cinnamic acid, caffeic acid, ferulic acid, chlorogenic acid, *p*-coumaric acid, and gallic acid derivatives. The highest proportion of flavonoids was attributed to luteolin, kaempferol, quercetin, and their derivatives, which corroborated our results. This included the presence of quercetin (up to 124 μg/g de) and its derivatives rutin, quercetin 3–*O*–Glc + Gal, and quercitrin (up to 64.2, 367, and 73 μg/g de, respectively), as well as high quantities of kaempferol 3–*O*–Glc. The presence of high amounts of rutin was also confirmed by Bozhuyuk et al. [31]. The substantial levels of the flavanol catechin and its derivative epicatechin in rosehips, recorded by Demir et al. [46], aligned with our findings, especially regarding catechin, which reached up to 347 μg/g de. The highest number of phenolic compounds was detected in cultivars BBA, ARA, and BA, which also exhibited the highest quantities for particular compounds. However, cultivar PAU ranked second in total phenolic content, making it another promising candidate. The cultivar RWA showed the lowest values.

Through a comparison between the rosehips investigated in this study and the rosehips of other *Rosa* genotypes, like wild roses *R. canina*, *Rosa arvensis*, *R. dumalis*, *Rosa dumentorum*, and *Rosa sempervirens* [5,12], it can be noted that the same flavonols and catechin were determined as the most abundant phenolic compounds. The dominancy varies, and future research could focus on establishing whether there are some traits that could be used as chemotaxonomic markers for certain species. It is also necessary to emphasize the effects of solvents on the quantities of the extracted compounds, as well as culinary techniques, i.e., processing technologies, that can also influence quantities of bioactive compounds [5,12].

The results on phenolic compounds content obtained herein align with the determined levels of antioxidant activity, which were higher for extracts of rosehips characterized by the highest amounts of phenolic compounds. This connection was confirmed by Ersoy et al. [48] in their assessment of *R. canina* rosehips.

‘Aurora’ rosehips, analyzed within this research, showed potent antioxidant activity. The results are in agreement with previously published results on wild rosehips. The IC_50_ values for the neutralization of DPPH^•^ reported for methanol extracts of air-dried rosehips in studies of others were in the range 11.8–81.1 and 14.2–117 μg/mL [5,12]. The same studies reported approximately 3.81–88.2 mg AAE/g de and 11.3–95.3 mg AAE/g de for FRAP assay. Fascella et al. [43] determined IC_50_ values in the range of 27.1–113.8 µg/mL in the DPPH assay for extracts of rosehips from four species grown in Sicily. Rosehips from the ‘Aurora’ collection showed good antioxidant activity that was comparable with the results obtained in other studies, with IC_50_ values in the range of 34.2–56.0 mg/mL; meanwhile, rosehips from the RWA cultivar showed lower antioxidant potential with IC_50_ value of 115 mg/mL for DPPH assay. In the FRAP assay, extracts of ‘Aurora’ rosehips showed values in the range of 37.5–68.5 mg AAE/g de which are comparable with other studies. Concerning the values obtained for synthetic antioxidant BHT, 9.32 μg/mL in DPPH assay and 124 mg AAE/g dw in FRAP assay, it can be concluded that ‘Aurora’ rosehips can serve as a source of antioxidants or can be added to food as additional protection from oxidation [5].

AChe has been identified as the leading therapeutic target for symptomatic treatment of Alzheimer’s disease [49]. Numerous compounds and plant extracts are screened in the search for designs of optimal inhibitory compounds. The extracts of rosehips and their preserves that were examined by Nađpal et al. [5] showed IC_50_ values in the range of 1.30–7.97 mg/mL, with the highest activities determined for methanol extracts. Extracts of rosehips examined in this study achieved 29.6–38.8% at a concentration of 0.5 mg/mL, which is comparable with methanol wild rosehip extracts examined by Nađpal et al. [5]. In the study by Olech and collaborators [50], rosehip tea and tincture showed AChE inhibition of 38.3 and 39%, respectively, at the concentration of 3.08 mg/mL. Nicolescu et al. [51] recorded IC_50_ values for rosehip extracts in the range of 1.09–7.50 mg/mL, which is comparable with rosehips examined in this study. This study testifies to the moderate inhibitory potential of ‘Aurora’ rosehips towards AChE.

A strong correlation exists between chemical composition and antioxidant activity, with Vit C and phenolic compounds identified as effective natural antioxidants. The DPPH assay indicated a high correlation with antioxidant activity for Vit C, TPC, TFC, TSC, quinic acid, and quercitrin. In contrast, the FRAP assay highlighted TPC, TFC, TSC, quinic and *p*-coumaric acids, quercetin, quercetin 3–*O*–Glc + Gal, quercitrin, and kaempferol 3–*O*–Glc as key contributors. Additionally, for AChE inhibition, TPC, TFC, TSC, and the presence of quinic acid, gallic acid, quercetin 3–*O*–Glc + Gal, quercitrin, and kaempferol 3–O–Glc demonstrated significant correlations.

## 5. Conclusions

This study explored the potential of garden rose rosehips as functional food ingredients, focusing on their morphological traits, phenolic profile, and biological activities. Roses, traditionally used for landscaping, are emerging as a promising source of functional food. However, there are limited data on the quality of garden rose rosehips, particularly regarding their phenolic content and biological effects. Our findings reveal that rosehips from the ‘Aurora’ collection exhibit high fruit mass, favorable mesocarp-to-fruit ratios, and promising morphological traits for both food processing and landscaping. Comparative analysis shows that these rosehips have significant levels of phenolic compounds and antioxidant activity, aligning with or surpassing results from other studies. Specifically, the ‘Aurora’ rosehips demonstrate potent antioxidant properties and a moderate inhibitory effect on AChE, which is relevant for Alzheimer’s disease research. These activities appear to be strongly correlated with Vit C and phenolics, which are greatly recognized as effective natural bioactive compounds.

Further research is essential to fully harness these benefits and position garden rose rosehips as a viable alternative to synthetic antioxidants. The insights from this study could enhance the marketability of garden roses, showcasing their potential not only as decorative plants but also as a source of rosehips for the production of functional and value-added foods.

## Figures and Tables

**Table 1:** Morphometric characterization of rosehips from ‘Aurora’ collection rose cultivars.

Cultivar	Fruit Mass (g)	Fruit Height (mm)	Fruit Width (mm)	Fruit Thickness (mm)	Mesocarp Mass (g)	Mesocarp Thickness (mm)	Seed Number per Fruit	Seed Mass (g)	Mesocarp-to-Fruit Ratio (%)
PAU	3.87 b	12.85 c	17.55 b	17.66 b	3.00 b	2.95 a	40.83 a	0.0135 a	76.89 a
BBA	4.40 ab	20.72 b	20.81 ab	20.44 ab	3.32 ab	3.15 a	56.17 a	0.0137 a	75.47 a
ARA	4.04 ab	17.79 b	19.50 ab	18.55 b	3.08 b	3.06 a	39.50 a	0.0136 a	76.13 a
BA	5.15 a	27.86 a	23.66 a	23.82 a	4.09 a	3.03 a	45.17 a	0.0142 a	79.5 a
RWA	3.96 b	14.22 c	17.17 b	17.53 b	3.05 b	3.00 a	35.18 a	0.0135 a	77.02 a

PAU—‘Purple Aurora’; BBA—‘Berry Bush Aurora’; ARA—‘Aromatic Aurora’; BA—‘Butterfly Aurora’; RWA—‘Rugose White Aurora’. Means within each column designated with different letters (a–c) differ significantly according to one-factor ANOVA followed by Tukey’s HSD test (*p* ≤ 0.05).

**Table 2:** Organoleptic traits of rosehips from the ‘Aurora’ rose collection.

Cultivar	Fruit Shape	Fruit Color	Mesocarp Color	Taste
PAU	Pitcher-shaped, flattened 100% *	Red	Orange	Medium acidity, medium sweetness
BBA	Pitcher-shaped, flattened 25%	Red	Orange	Medium acidity, medium sweetness
ARA	Pitcher-shaped, flattened 50%	Red	Orange	Medium acidity, medium sweetness
BA	Pitcher-shaped, not flattened	Orange	Orange	Medium acidity, medium sweetness
RWA	Pitcher-shaped, flattened 75%	Orange	Orange	Medium acidity, medium sweetness

PAU—‘Purple Aurora’; BBA—‘Berry Bush Aurora’; ARA—‘Aromatic Aurora’; BA—‘Butterfly Aurora’; RWA—‘Rugose White Aurora’. * Proportion of flattened fruits out of the total number of assessed fruits for a particular cultivar.

**Table 3:** Contents of total phenols (TPC), total flavonoids (TFC), and vitamin C (Vit C) in rosehip extracts.

Cultivar	TPC		TFC		Vit C
	mg GAE/g de	mg GAE/g fw	mg QE/g de	µg QE/g fw	µg/g fw
PAU	55.7 ± 3.55 b	7.72 ± 0.49 a	2.14 ± 0.09 a	296 ± 12.1 a	2348 ± 121 a
BBA	63.9 ± 1.84 a	6.45 ± 0.19 b	1.80 ± 0.05 b	182 ± 4.74 c	2135 ± 57.0 b
ARA	47.4 ± 1.25 c	4.57 ± 0.12 d	0.85 ± 0.01 d	82.1 ± 1.09 e	2384 ± 0.87 a
BA	58.6 ± 2.77 ab	5.34 ± 0.25 c	1.12 ± 0.10 c	102 ± 9.10 d	2011 ± 36.1 b
RWA	37.1 ± 0.99 d	4.75 ± 0.13 d	1.70 ± 0.10 b	218 ± 12.9 b	218 ± 8.88 c

PAU—‘Purple Aurora’; BBA—‘Berry Bush Aurora’; ARA—‘Aromatic Aurora’; BA—‘Butterfly Aurora’; RWA—‘Rugose White Aurora’. GAE—gallic acid equivalents; QE—quercetin equivalents; de—dry extract; fw—fresh weight. Means designated with different letters (a–e) differ significantly according to one-factor ANOVA followed by Tukey’s HSD test (*p* ≤ 0.05).

**Table 4:** Contents of quinic acid and selected phenolic compounds in rosehip methanol extracts.

Cultivar	Content [μg/g de] *				
	PAU	BBA	ARA	BA	RWA
Quinic acid	**176** ** ± 17.6 ac	**132** ± 13.2 bc	**85.9** ± 8.59 d	**161** ± 16.1 ab	**116** ± 11.6 cd
Hydroxybenzoic acids					
*p*-Hydroxybenzoic acid	1.35 ± 0.08 b	1.63 ± 0.10 a	1.83 ± 0.11 a	1.73 ± 0.10 a	0.79 ± 0.05 c
Gentisic acid	nd ***	0.49 ± 0.04 a	0.58 ± 0.05 a	<0.15 ****	nd
Protocatechuic acid	4.81 ± 0.39 a	5.70 ± 0.46 a	5.45 ± 0.44 a	2.70 ± 0.22 b	2.58 ± 0.21 b
Gallic acid	**28.0** ± 2.52 c	**46.6** ± 4.20 b	**30.3** ± 2.73 c	**30.3** ± 2.72 c	**63.4** ± 5.71 a
Syringic acid	1.23 ± 0.25 ab	0.65 ± 0.13 b	1.31 ± 0.26 a	1.31 ± 0.26 a	nd
Hydroxycinnamic acids					
*p*-Coumaric acid	**18.5** ± 1.66 c	**46.7** ± 4.20 a	**18.4** ± 1.66 c	**34.8** ± 3.13 b	**14.5** ± 1.31 c
Caffeic acid	5.02 ± 0.35 c	**10.6** ± 0.74 a	9.60 ± 0.67 a	7.82 ± 0.55 b	2.98 ± 0.21 b
Ferulic acid	1.55 ± 0.16 cd	2.38 ± 0.24 b	2.00 ± 0.20 bc	3.07 ± 0.31 a	1.25 ± 0.12 d
Sinapic acid	nd	nd	<0.60	<0.60	0.89 ± 0.09
Chlorogenic acid	0.46 ± 0.02 c	0.97 ± 0.05 b	2.21 ± 0.11 a	2.39 ± 0.12 a	0.53 ± 0.03 c
Coumarins					
Esculetin	0.20 ± 0.01 b	0.20 ± 0.01 b	0.44 ± 0.03 a	0.15 ± 0.01 c	0.15 ± 0.01 c
Flavonols					
Quercetin	**52.4** ± 15.7 b	**124** ± 37.1 a	**106** ± 31.8 ab	**80.4** ± 24.1 ab	**48.5** ± 14.6 b
Rutin	**36.0** ± 1.08 c	**64.2** ± 1.93 a	**48.8** ± 1.46 b	**29.3** ± 0.88 d	**12.4** ± 0.37 e
Quercetin 3–*O*–Glc + Gal	**234** ± 14.0 c	**367** ± 22.0 a	**261** ± 15.7 bc	**291** ± 17.5 b	**343** ± 20.6 a
Quercitrin	**42.5** ± 2.55 c	**57.7** ± 3.46 b	**45.9** ± 2.75 c	**73.0** ± 4.38 a	**29.2** ± 1.75 d
Isorhamnetin	2.23 ± 0.13 a	<1.20	1.39 ± 0.08 b	<1.20	1.31 ± 0.08 b
Kaempferol	5.98 ± 0.42 b	5.62 ± 0.39 b	8.02 ± 0.56 a	5.80 ± 0.41 b	5.59 ± 0.39 b
Kaempferol 3–*O*–Glc	**40.9** ± 1.63 b	**37.9** ± 1.51 b	**46.8** ± 1.87 a	**37.9** ± 1.52 b	**37.9** ± 1.52 b
Flavones					
Amentoflavone	<4.9	<4.9	<4.9	<4.9	<4.9
Luteolin	<0.60	1.27 ± 0.06	<0.60	<0.60	<0.60
Luteolin 7–*O*–Glc	3.25 ± 0.10 c	4.14 ± 0.12 a	1.26 ± 0.04 d	1.28 ± 0.04 d	3.56 ± 0.11 b
Apigenin	<0.3	<0.3	<0.3	<0.3	<0.3
Apigenin 7–*O*–Glc	0.15 ± 0.01 bc	0.40 ± 0.02 a	0.13 ± 0.01 c	0.23 ± 0.01 b	nd
Vitexin	nd	0.19 ± 0.01	nd	nd	nd
Apiin	nd	0.21 ± 0.01	nd	nd	nd
Chrysoeriol	1.45 ± 0.04 a	1.16 ± 0.03 b	0.75 ± 0.02 d	1.06 ± 0.03 c	<0.60
Myricetin	nd	<19.6	<19.6	<19.6	<19.6
Flavanones					
Naringenin	3.13 ± 0.22 c	6.78 ± 0.47 a	5.57 ± 0.39 b	6.61 ± 0.46 a	1.64 ± 0.11 d
Flavanols					
Catechin	**347** ± 34.7 a	**279** ± 27.9 b	**146** ± 14.6 c	**94.2** ± 9.42 cd	**43.0** ± 4.30 d
Epicatechin	5.84 ± 0.58 a	3.43 ± 0.34 b	2.22 ± 0.22 c	2.87 ± 0.29 bc	0.58 ± 0.06 d
Total phenolics [mg/g de] *****	1.01	1.20	0.83	0.87	0.73

PAU—‘Purple Aurora’; BBA—‘Berry Bush Aurora’; ARA—‘Aromatic Aurora’; BA—‘Butterfly Aurora’; RWA—‘Rugose White Aurora’. Means within each row with different letters (a–e) differ significantly according to one-factor ANOVA followed by Tukey’s HSD test (*p* ≤ 0.05); * results are given as content (µg/g of dry extract) ± standard error of repeatability (as determined via method validation); ** values higher than 10 are marked with bold letters; *** not detected; **** below the limit of quantification (LOQ); ***** sum of the contents of all detected compounds using LC-MS/MS.

**Table 5:** Antioxidant activity and potential of rosehip extract to inhibit AChE.

Cultivar	IC_50_ (DPPH)	FRAP	AChE
	µg/mL	mg AAE/g de	% of Inhibition
PAU	34.5 ± 1.47 c	52.6 ± 3.40 c	38.8 ± 1.25 a
BBA	34.2 ± 2.47 c	68.5 ± 0.51 a	38.3 ± 3.14 a
ARA	56.0 ± 3.17 b	37.7 ± 1.76 d	29.6 ± 1.48 b
BA	37.4 ± 1.11 c	56.6 ± 0.46 b	37.2 ± 4.00 a
RWA	115 ± 9.16 a	37.5 ± 0.04 d	40.4 ± 1.80 a

PAU—‘Purple Aurora’; BBA—‘Berry Bush Aurora’; ARA—‘Aromatic Aurora’; BA—‘Butterfly Aurora’; RWA—‘Rugose White Aurora’; IC_50_ (DPPH)—the concentration of the extract that neutralizes 50% of DPPH radicals; FRAP—Ferric reducing antioxidant potential; AAE—ascorbic acid equivalents; AchE—acetylcholine esterase; de—dry extract. Means within each row with different letters (a–d) differ significantly according to one-factor ANOVA followed by Tukey’s HSD test (*p* ≤ 0.05).

**Table 6:** Correlation factors (r^2^) between chemical composition and biological activities.

	DPPH	FRAP	AChE
Vit C	0.940	0.866	0.785
TPC	0.968	0.991	0.960
TFC	0.856	0.913	0.950
TSC	0.950	0.988	0.966
Quinic acid	0.936	0.954	0.965
*p*-Coumaric acid	0.899	0.942	0.827
Gallic acid	0.672	0.824	0.919
Quercetin 3–*O*–Glc + Gal	0.841	0.946	0.977
Quercitrin	0.938	0.954	0.901
Rutin	0.887	0.885	0.785
Quercetin	0.862	0.907	0.844
Kaempferol 3–*O*–Glc	0.872	0.921	0.967
Catechin	0.871	0.799	0.720

Vit C—vitamin C content; TPC—total phenolic content; TFC—total flavonoid content; TSC—total sum of compounds quantified by LC-MS/MS.

## Data Availability

The original contributions presented in the study are included in the article/Supplementary Materials, further inquiries can be directed to the corresponding author.

## Supplementary Materials

The following supporting information can be downloaded at: https://www.mdpi.com/article/10.3390/foods13203272/s1, Table S1: LC-MS/MS parameters and standard curve equations for all quantified compounds in rosehip methanol extracts; Figure S1: TIC chromatogram of PAU methanol extract obtained in MRM mode; Figure S2: TIC chromatogram of BBA methanol extract obtained in MRM mode; Figure S3: TIC chromatogram of ARA methanol extract obtained in MRM mode; Figure S4: TIC chromatogram of BA methanol extract obtained in MRM mode; Figure S5: TIC chromatogram of RWA methanol extract obtained in MRM mode.
