# Supplementary material for: In Vitro Functional Properties of Rosehips from ‘Aurora’ Edible Garden Rose’s Collection
Source: Foods. 2024 Oct 15;13(20):3272. doi: 10.3390/foods13203272 (PMC11507501; doi:10.3390/foods13203272)
Supplement: Supplementary file 1 [file foods-13-03272-s001.zip › foods-3232970-supplementary.pdf]

foods

MDPI

Supplementary Materials

In Vitro Functional Properties of Rosehips from 'Aurora' Edible Garden Rose's Collection

Nemanja Živanović [1], Biljana Božanić Tanjga [2], Nataša Simin [1], Marija Lesjak [1], Bojana Blagojević [3], Magdalena Pušić Devai [3], Mirjana Ljubojević [3,*] and Tijana Narandžić [3]

[1] Faculty of Sciences, University of Novi Sad, Trg Dositeja Obradovića 3, 21000 Novi Sad, Serbia; nemanja.zivanovic@dh.uns.ac.rs (N.Ž.); natasa.simin@dh.uns.ac.rs (N.S.); marija.lesjak@dh.uns.ac.rs (M.L.)
[2] Breeding Company 'Pheno Geno Roses', Maršala Tita 75, 23326 Ostojićevo, Serbia; b.bozanic@phenogenoroses.com
[3] Faculty of Agriculture, University of Novi Sad, Trg Dositeja Obradovića 8, 21000 Novi Sad, Serbia; bojana.blagojevic@polj.uns.ac.rs (B.B.); magdalena.pusic@polj.uns.ac.rs (M.P.D.); tijana.narandzic@polj.uns.ac.rs (T.N.)
* Correspondence: ikrasevm@polj.uns.ac.rs; Tel.: +381-214853251

Table S1. LC-MS/MS parameters and standard curve equations for all quantified compounds in rosehip methanol extracts.

Compound	t_R [min]	Precursor m/z	Product m/z	$V_{fragmentor}$ [V]	$V_{collision}$ [V]	Standard Curve Equation	Coefficient of Determination r^2
Organic acids							
Quinic acid	0.52	191	85	150	20	y = 43.05882 + 0.99275*x + (-5.8E-5)*x^2	0.99826
Hydroxybenzoic acids							
p-Hydroxybenzoic acid	1.13	137	93	80	10	y = 26 + 7.54721*x	0.99684
Protocatechuic acid	0.81	153	109	105	9	y = 76.21393 + 8.4621*x	0.99808
Gentisic acid	1.04	153	109	100	9	y = 16.79167 + 6.18408*x	0.9949
Gallic acid	0.61	169	125	90	10	y = 313.50981 + 7.24903*x + (-5.28143E-4)*x^2	0.99722
Syringic acid	1.32	197	182	90	7	y = -4.29412 + 2.0345*x + (-0.00139)*x^2	0.99683
Hydroxycinnamic acids							
p-Coumaric acid	1.73	163	119	90	9	y = 237.48334 + 14.87448*x + (-0.00302)*x^2	0.99921
Caffeic acid	1.19	179	135	100	10	y = 121.57711 + 14.82432*x	0.99773
Ferulic acid	1.93	193	134	90	11	y = 11.40299 + 3.4648*x	0.9985
Sinapic acid	1.91	223	193	100	17	y = -5.95652 + 1.56645*x	0.98915
Chlorogenic acid	0.84	353	191	100	10	y = 25.78109 + 14.68482*x	0.99368
Coumarins							
Esculetin	1.15	177	133	105	15	y = 2.09453 + 11.62414*x	0.99839
Flavanols							
Catechin	0.75	289	245	150	10	y = 117.11575 + 0.87116*x + (-3.19065E-5)*x^2	0.99855
Epicatechin	1.02	289	245	150	10	y = 7.08333 + 1.6188*x	0.99323
Flavanones							

Compound	t_R [min]	Precursor m/z	Product m/z	$V_{fragmentor}$ [V]	$V_{collision}$ [V]	Standard Curve Equation	Coefficient of Determination r^2
Naringenin	3.93	271	151	130	16	y = 21.12438 + 8.64614*x	0.99965
				Flavones			
Luteolin	4.05	285	133	135	25	y = -11.83333 + 8.80288*x	0.99446
Luteolin 7–*O*–Glc	2.23	447	285	230	30	y = 87.5 + 27.97304*x	0.99864
Apigenin 7–*O*–Glc	2.78	431	268	135	41	y = 27.375 + 25.92107*x	0.99995
Vitexin	1.96	431	311	200	22	y = 21.41667 + 16.27654*x	0.989
Apiin	2.65	563	269	250	36	y = 13.20833 + 25.34675*x	0.99573
				Flavonols			
Quercetin	3.78	301	151	130	15	y = 2093.91304 + 6.55124*x	0.99647
Rutin	2.27	609	300	135	42	y = 137.1092 + 7.06661*x	0.99879
Quercetin 3–*O*–Glc	2.33	463	300	210	30	y = 3922.33333 + 8.51734*x	0.99784
Quercetin 3–*O*–Gal	2.33	463	300	200	30	y = 3922.33333 + 8.51734*x	0.99784
Quercitrin	2.87	447	300	190	27	y = 593.29167 + 12.51799*x	0.99952
Isorhamnetin	4.86	315	300	160	21	y = -64.86957 + 18.02917*x	0.99273
Kaempferol	4.61	285	285	130	0	y = 115.29167 + 44.77264*x	0.99996
Kaempferol 3–*O*–Glc	2.91	447	284	190	30	y = 486.04167 + 6.93922*x	0.99928

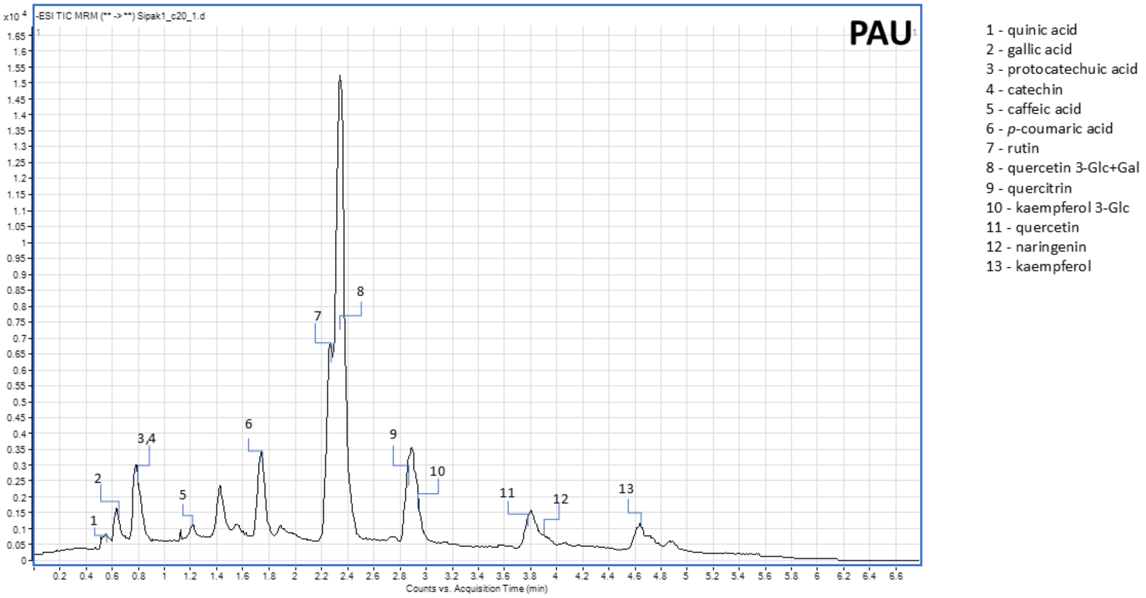

Figure S1. TIC chromatogram of PAU methanol extract obtained in MRM mode.

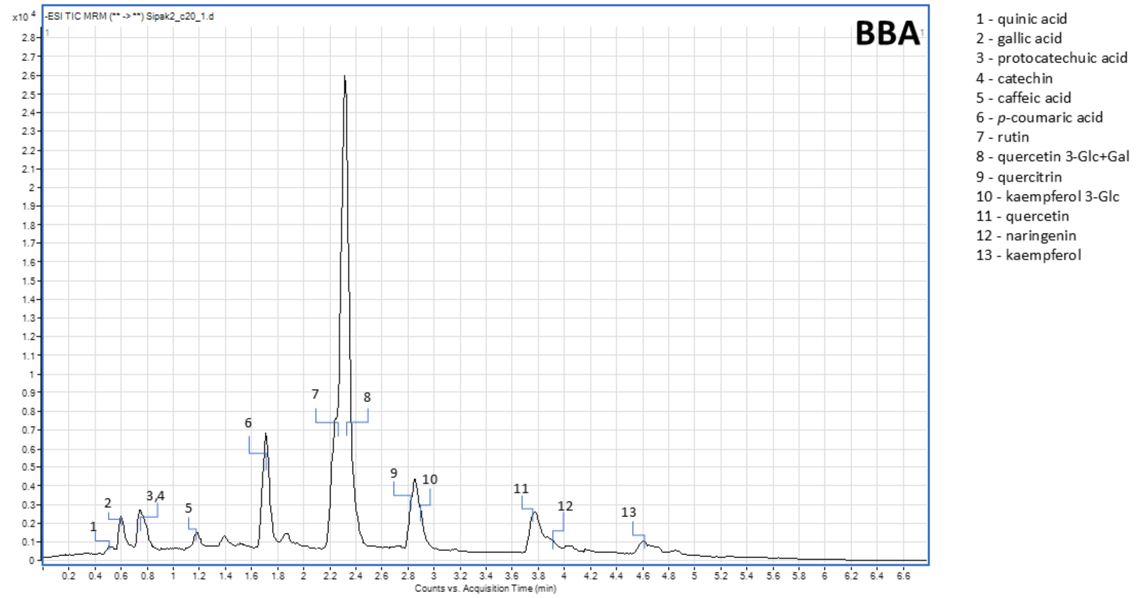

Figure S2. TIC chromatogram of BBA methanol extract obtained in MRM mode.

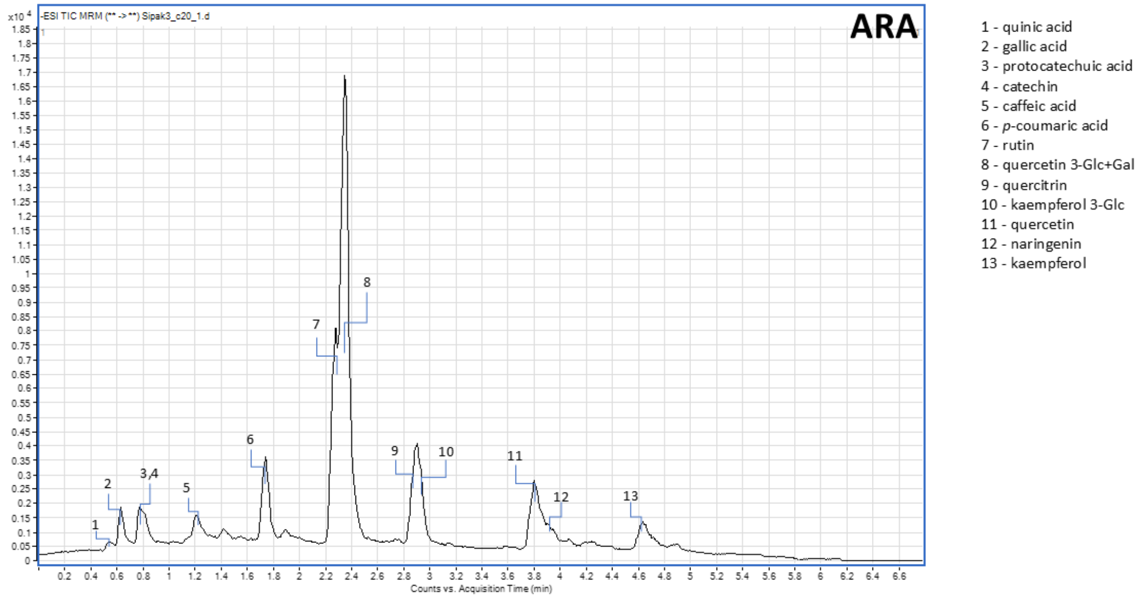

Figure S3. TIC chromatogram of ARA methanol extract obtained in MRM mode.

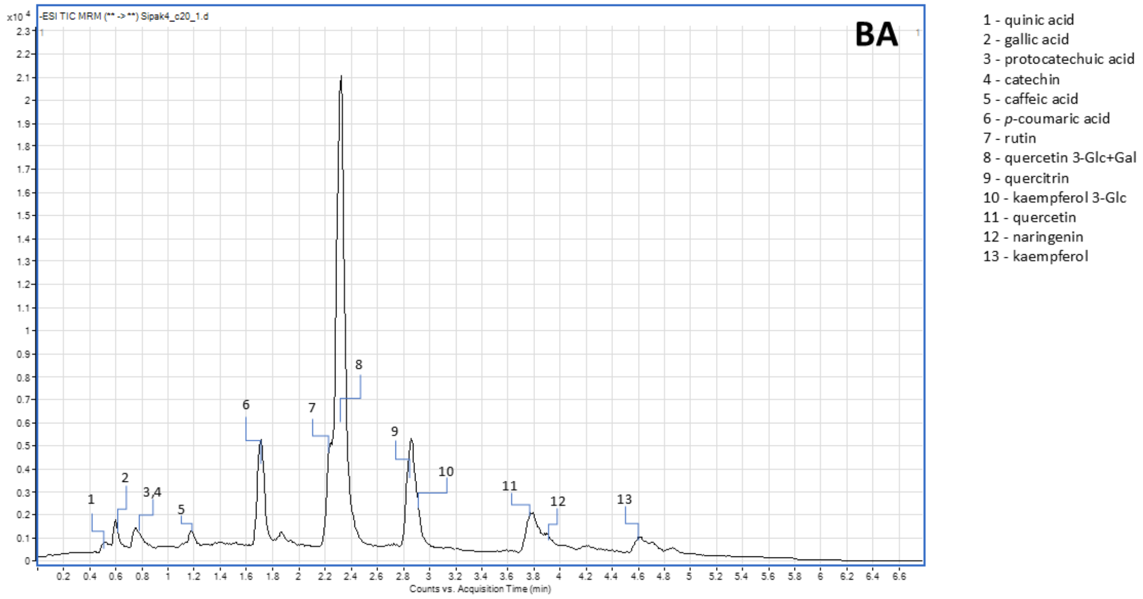

Figure S4. TIC chromatogram of BA methanol extract obtained in MRM mode.

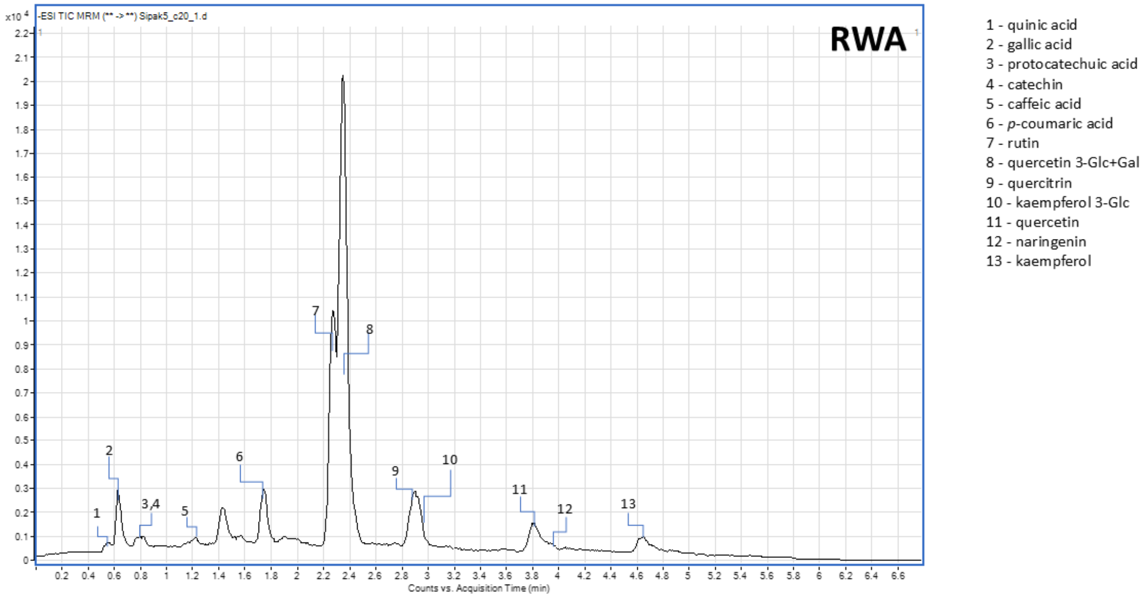

Figure S5. TIC chromatogram of RWA methanol extract obtained in MRM mode.
